# Identification of senescent cell subpopulations by CITE‐seq analysis

**DOI:** 10.1111/acel.14297

**Published:** 2024-08-14

**Authors:** Kotb Abdelmohsen, Krystyna Mazan‐Mamczarz, Rachel Munk, Dimitrios Tsitsipatis, Qiong Meng, Martina Rossi, Apala Pal, Chang Hoon Shin, Jennifer L. Martindale, Yulan Piao, Jinshui Fan, Hagai Yanai, Supriyo De, Isabel Beerman, Myriam Gorospe

**Affiliations:** ^1^ Laboratory of Genetics and Genomics National Institutes of Health (NIH) Baltimore Maryland USA; ^2^ Translational Gerontology Branch National Institute on Aging (NIA) Intramural Research Program (IRP), National Institutes of Health (NIH) Baltimore Maryland USA

**Keywords:** cell cycle, CITE‐seq, proteome, senescence, single‐cell transcriptome, surface proteins, surfaceome

## Abstract

Cellular senescence, a state of persistent growth arrest, is closely associated with aging and age‐related diseases. Deciphering the heterogeneity within senescent cell populations and identifying therapeutic targets are paramount for mitigating senescence‐associated pathologies. In this study, proteins on the surface of cells rendered senescent by replicative exhaustion and by exposure to ionizing radiation (IR) were identified using mass spectrometry analysis, and a subset of them was further studied using single‐cell CITE‐seq (Cellular Indexing of Transcriptomes and Epitopes by Sequencing) analysis. Based on the presence of proteins on the cell surface, we identified two distinct IR‐induced senescent cell populations: one characterized by high levels of CD109 and CD112 (cluster 3), the other characterized by high levels of CD112, CD26, CD73, HLA‐ABC, CD54, CD49A, and CD44 (cluster 0). We further found that cluster 0 represented proliferating and senescent cells in the G1 phase of the division cycle, and CITE‐seq detection of cell surface proteins selectively discerned those in the senescence group. Our study highlights the heterogeneity of senescent cells and underscores the value of cell surface proteins as tools for distinguishing senescent cell programs and subclasses, paving the way for targeted therapeutic strategies in disorders exacerbated by senescence.

## Graphical abstract

The surfaceome of senescent cells was studied by mass spectrometry analysis. CITE‐seq (Cellular Indexing of Transcriptomes and Epitopes by Sequencing) analysis was then performed using antibodies that recognized proteins specifically abundant on the surface of senescent cells. Our analysis revealed that specific surface proteins identified distinct subsets of senescent cells, paving the way for therapeutic strategies targeting select senescent cell subpopulations.

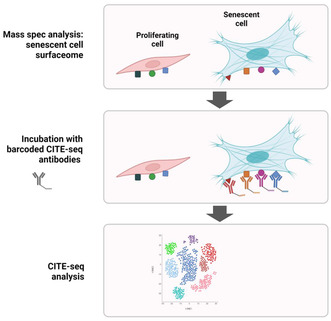

**Abbreviations**
CITE‐seq: cellular indexing of transcriptomes and epitopes by sequencing
IRIS: ionizing radiation‐induced senescence
PDL: population doubling level
RS: replicative senescence
SASP: senescence‐associated secretory phenotype
SA‐β‐Gal: senescence‐associated‐β‐galactosidase

## 1 INTRODUCTION

Cellular senescence is a state of enduring cell cycle arrest that occurs in response to sublethal damage from sources internal and external to the cell (Herranz & Gil, 2018; Wiley & Campisi, 2016). Senescent cells exhibit distinct characteristics such as altered gene expression programs, resistance to apoptosis, enlarged and flattened cell morphology, dysfunctional organelles, and increased lysosomal function with elevated senescence‐associated β‐galactosidase (SA‐β‐Gal) activity. Furthermore, senescent cells secrete proinflammatory cytokines, growth factors, and tissue‐remodeling enzymes; this trait is known as the senescence‐associated secretory phenotype (SASP) and it strongly influences tissue function through inflammation and fibrosis (Hernandez‐Segura et al., 2018; Salama et al., 2014; Yosef et al., 2016).

Senescent cells accumulate in tissues and organs as aging progresses, contributing to age‐related disability and disease (Childs et al., 2015; van Deursen, 2014). Consequently, targeting senescent cells through selective removal using senolytic drugs has shown promise in extending health span and improving age‐associated disorders in animals (Chaib et al., 2022; Sun et al., 2022). Cellular senescence is a heterogeneous process, and this heterogeneity poses a major challenge for senescent cell characterization and effective senolysis (Gonzalez‐Gualda et al., 2021; Hernandez‐Segura et al., 2018; Sharpless & Sherr, 2015). In this regard, proteins on the surface of senescent cells are increasingly leveraged to overcome these challenges and subclassify senescent cell populations (Rossi & Abdelmohsen, 2021). We previously identified DPP4 (dipeptidyl peptidase 4, also known as CD26) and SCAMP4 (secretory carrier membrane protein 4) as being highly expressed on the surface of senescent cells and thereby providing access for interventions to eliminate senescent cells (Kim et al., 2017, 2018). The heightened presence of DPP4 rendered senescent cells targetable for elimination, while SCAMP4 actively promoted the secretion of SASP factors and thus was a promising target of senomorphic interventions (Herman et al., 2023; Kim et al., 2018). Similarly, other senescent cell surface proteins also represent opportunities for senolysis and/or senotherapy (Rossi & Abdelmohsen, 2021).

Single‐cell RNA sequencing (scRNA‐seq) analysis has revolutionized the field of cell biology by recognizing cell heterogeneity within a population through profiling the transcriptomes of individual cells (Choi & Kim, 2019). Accordingly, scRNA‐seq analysis has helped to uncover the rich diversity of cells in various disease contexts, including immune diseases, cancers, and infections (Gonzalez‐Silva et al., 2021; SoRelle et al., 2021; Xu et al., 2022). However, cell transcriptomes and proteomes do not always match in different cellular processes, including cell senescence (Casella et al., 2019; Srikantan et al., 2011); for instance, the senescence‐associated proteins p16 (CDKN2A) and p53 (TP53) are more robust indicators of senescence than the corresponding mRNAs (Casella et al., 2019). Broadly speaking, the discrepancies between mRNA and protein levels arise from regulatory events like alternative splicing, altered mRNA export and stability, and changes in translation and protein processing. These processes are influenced by a wide range of RNA‐binding proteins and noncoding RNAs (Pozo et al., 2021). Unfortunately, complementary methods such as single‐cell proteomic analyses are still in their infancy and cannot reliably inform on protein content at the level of an individual cell.

To begin to harmonize scRNA‐seq analysis with the proteins expressed in a given cell, a few methodologies have been developed in recent years. One of these technologies, CITE‐seq (Cellular Indexing of Transcriptomes and Epitopes by Sequencing) analysis, employs a combination of scRNA‐seq and cell‐surface protein analysis using specialized barcoded antibodies to label and quantify cell‐surface proteins (Cohn et al., 2023). This approach permits a direct link between the scRNA profiles and the expression of cell‐surface proteins. Thus, CITE‐seq is particularly attractive for studies of cellular heterogeneity in senescence, where connecting transcriptomes with key proteins expressed on the cell surface is critical for understanding different functional states of the cell.

In this proof‐of‐principle study, we hypothesized that senescence‐associated surface proteins can help distinguish specific subsets of senescent cells defined by distinct transcriptomes identified by scRNA‐seq analysis. To test this idea, we first identified cell‐surface proteins associated with senescence, and then utilized barcoded antibodies recognizing these proteins for subsequent CITE‐seq analysis. Among the senescent cell subpopulations identified as having distinct transcriptomes, two cell clusters warranted further study, as they also preferentially expressed surfaceome markers identified by CITE‐seq analysis. One subpopulation displayed high cell‐surface levels of CD109 and CD112 proteins (cluster 3), the other subpopulation exhibited high cell‐surface levels of CD112, CD26, CD73, HLA‐ABC, CD54, CD49A, and CD44 proteins (cluster 0). Both subpopulations were extremely abundant in cells rendered senescent by IR, and both expressed increased levels of multiple mRNAs encoding inflammatory factors. However, within cluster 0, which comprised many proliferating and senescent cells in the G1 phase of the division cycle, CITE‐seq detection of membrane markers allowed the selective identification of senescent cells. In sum, we report the value of cell‐surface proteins to distinguish and classify distinct senescent cell subpopulations in order to devise future targeted interventions that alleviate the detrimental effects of senescence in pathologic states.

## 2 RESULTS

### 2.1 Analysis of the senescence‐associated surfaceome

**FIGURE 1:**
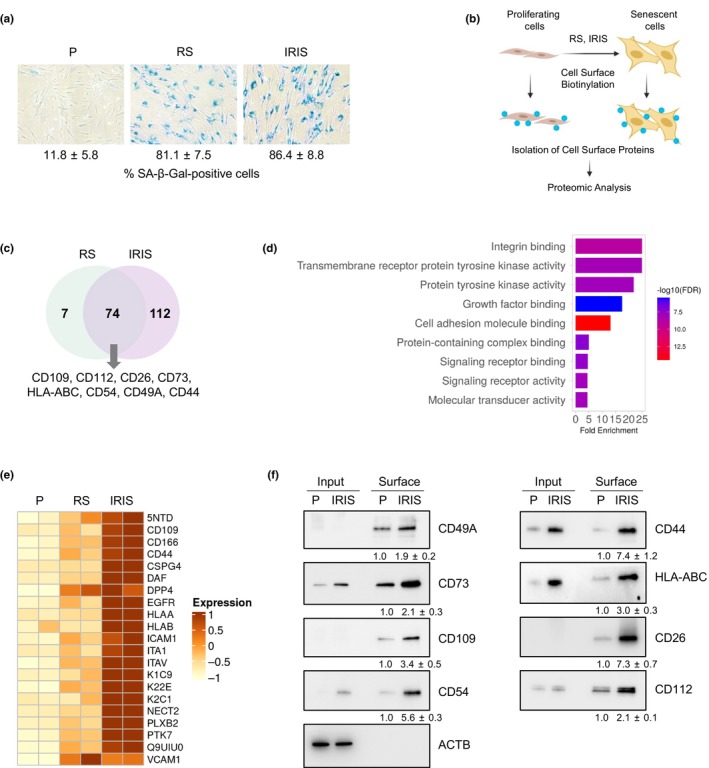
Uncovering the senescence‐associated surface proteome. (a) SA‐β‐Gal activity was analyzed in WI‐38 fibroblasts at ~PDL24 (proliferating, P), after reaching replicative exhaustion at ~PDL54 (RS), or after exposure to 10 Gy of ionizing radiation and culture for an additional 10 days (IRIS). (b) Workflow for cell‐surface protein biotinylation and isolation for proteomic analysis; prepared using BioRender. (c) Overlap of the cell surface proteins elevated in RS vs P (green) and IRIS vs P (pink) after processing as in panels (a, b). (d) Top significantly (FDR <0.05) enriched GO Molecular Function terms after ShinyGO analysis performed on the shared cell surface proteins upregulated in senescent cells. (e) Heatmap illustrating the magnitude of induction in a subset of surface proteins, comparing RS to P cells and IRIS to P cells that were prepared as explained in (a). In the heatmap, 5NTD, ICAM1, DPP4, NECT2, and ITA1 are CD73, CD54, CD26, CD112, and CD49A, respectively. (f) WI‐38 cells were processed as in (a), and the levels of CD49A, CD73, CD109, CD54, CD112, CD44, HLA‐ABC, CD26, and loading control β‐Actin (ACTB) were assessed in Input (whole‐cell proteins) and Surface (cell‐surface proteins) by western blot analysis. “Input”, 10 μg of the protein lysate before pulldown; “Surface”, 5% of the eluted protein volume after pulldown. As the lysis buffer did not fully solubilize membrane proteins, only “Surface” proteins are quantified (ImageJ). Data are representative of three independent replicates.

To establish senescence, WI‐38 human diploid fibroblasts that were proliferating (P) (population doubling level [PDL] ~24) were cultured until their replicative potential was exhausted (~PDL54) and they reached replicative senescence (RS), or were exposed to 10 Gy of ionizing radiation (IR) and cultured for an additional 10 days. Cell senescence was confirmed by evaluating senescence‐associated (SA)‐β‐Gal activity, which was visualized as a blue stain (Figure 1a); senescence was also confirmed by assessing the levels of RNA markers that increase with senescence (*p21*, *CXCL1*, *GDF15*, *BAFF*, *DPP4*, and *IL1A* mRNAs, as well as the long noncoding RNA *PURPL*) or decrease with senescence (*MKI67* and *LMNB1* mRNAs) (Figure S1). Cell surface proteins were then labeled using cell surface biotinylation and were isolated (Figure 1b) for mass spectrometry (MS) analysis to identify the senescence‐associated surface proteome in each paradigm. MS analysis revealed significant alterations in the senescence‐associated surface proteome (the “surfaceome”) in both senescence models compared to proliferating cells (Table S1), indicating that cell surface proteins were actively remodeled during senescence, with specific proteins increasing and decreasing on the cell surface. MS analysis followed by identification of differentially abundant surface proteins in each comparison group revealed proteins significantly upregulated in RS and IR‐induced senescence (IRIS), with a total of 74 shared proteins elevated in both comparison groups (Figure 1c). The functional significance of these proteins, as identified by Gene Ontology (GO) enrichment analysis using ShinyGO (Ge et al., 2020), revealed that the senescent cell surface proteins were associated with both integrin and growth factor binding, as well as with transmembrane activity (Figure 1d).

To validate the identified surface proteins, we focused on IRIS, which yielded stronger protein changes relative to RS (Figure 1e). Western blot analysis confirmed that proteins CD49A, CD73, CD109, CD54, CD112, CD44, HLA A/B/C, and CD26 were more abundant on the surface of senescent cells; ACTB (β‐Actin) was included as a loading control protein expressed in the input samples (Figure 1f). Together, these data indicate that the cell surface proteome, the “surfaceome”, changes robustly during senescence, and that several proteins are jointly upregulated in both senescence models.

### 2.2 Identification of cell subpopulations by transcriptomic analysis

**FIGURE 2:**
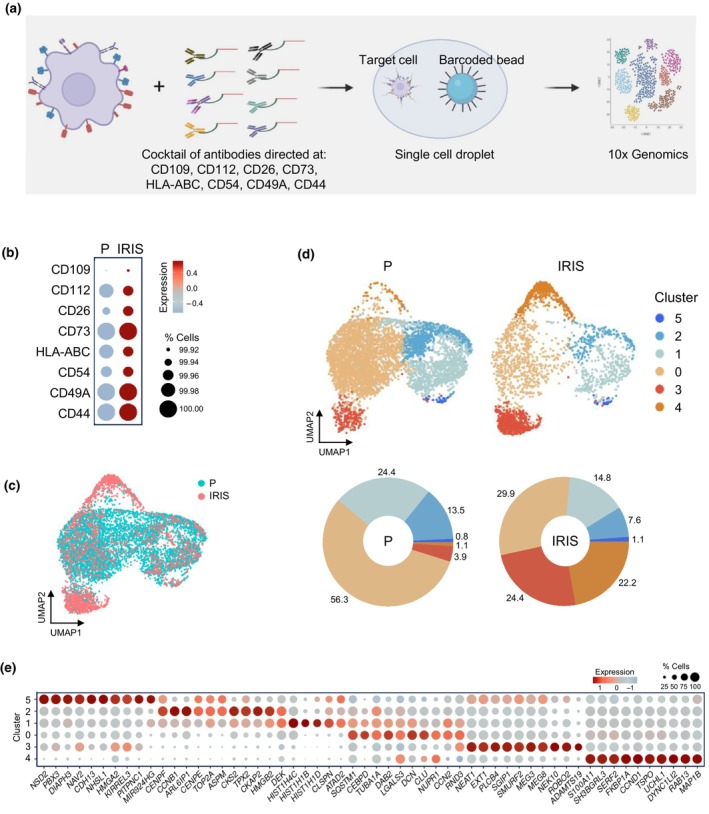
UMAP representation and clustering analysis of CITE‐seq. (a) Workflow illustrating the use of a cocktail of CITE‐seq antibodies to incubate with cells, followed by single‐cell sequencing analysis on the 10× Genomics platform. Illustration was created using BioRender. (b) ADT levels of cell‐surface proteins in cell populations that were either proliferating (P) or rendered senescent by exposure to IR and additional culture for 10 days (IRIS). Color indicates scaled average expression and dot size represents the percentage of cells expressing ADT. (c) UMAP of integrated samples, color‐labeled for P and IRIS cell populations. (d) UMAP illustrating the cell clusters (*top*), and percent composition of each cluster (*bottom*) in P and IRIS populations. (e) Genes (x‐axis) corresponding to the ten most highly expressed marker RNAs of each cluster. Color indicates average expression scaled across all clusters and dot size represents the percentage of cells expressing specific RNAs.

To better characterize the different senescent cell subpopulations, we sought to study the transcriptomic profiles and the corresponding surface protein expression patterns of individual cells. We employed the CITE‐seq methodology, which enables the simultaneous measurement of surface proteins and transcriptomes in single cells (Stoeckius et al., 2017). The robust and specific expression levels of surface proteins in senescent cells, as demonstrated by proteomic and western blot analyses (Figure 1), suggest that they have a strong potential to serve as senescence markers. As illustrated in the schematic (Figure 2a), we incubated special barcoded antibodies, each recognizing one of the eight senescence‐associated surface proteins validated in Figure 1f, with IRIS and P cells, and then carried out CITE‐seq analysis using the 10× Genomics platform (GSE250041). In this manner, for a given cell, the analysis included the endogenous single‐cell transcriptomes and antibody‐derived tags (ADTs) resulting from amplification of the barcodes on the antibodies recognizing the cell surface proteins present on that cell. Consistent with the proteomic data, CITE‐seq analysis confirmed the elevated levels of ADTs, indicating increased expression of all these proteins on the surface of IRIS cells compared to P cells (Figure 2b).

We then explored the transcriptomes of these cells by first integrating P and IRIS populations (Figure 2c), which revealed six clusters of cells with distinct transcriptomic patterns (Figure 2d). Within these clusters, the percentages of cells in clusters 0, 1, and 2 declined in IRIS compared to P populations, while clusters 3 and 4 increased in IRIS compared to P populations, with transcripts mapping to specific genes in each group. The genes represented by the transcriptomes of clusters 1 and 2, most abundant in P cells, encode proteins that promote cell proliferation and DNA replication, such as *CCNB1*, *TOP2A*, *CKS2*, *HMGB2*, and *CENPF* (Figure 2e; Table S2). In contrast, clusters 3 and 4, most abundant in IRIS cells (Figure 2d, *bottom*), displayed transcriptomes associated with the senescence phenotype; cluster 3 cells displayed high levels of the long noncoding RNAs *NEAT1*, induced by p53 and implicated in growth arrest and senescence (Mello et al., 2017; Pan et al., 2022), and *MEG3*, which inhibits cell proliferation and promotes the accumulation of G1‐phase cells (Luo et al., 2015), while cluster 4 displayed high levels of *CCND1* and *CDKN2A* mRNAs, encoding senescence‐associated proteins cyclin D1 (CCND1) and cyclin‐dependent kinase inhibitor p16 (CDKN2A), respectively. In addition, the transcriptomes of clusters 3 and 4 correlated significantly (*R* = 0.96, *R* = 0.91) with distinct states of senescence described earlier in a model of etoposide‐induced senescence (Wechter et al., 2023) (Figure S2b,c). The analysis also determined that cells of cluster 0, abundant in both P and IRIS populations, expressed low levels of mRNAs encoding pro‐proliferative proteins and shared transcriptomes widely with other clusters (Figure 2e), suggesting that cluster 0 comprises a mixture of cells in various states, as we elaborate below (section 2.5).

### 2.3 Expression of cell‐surface proteins in identified cell clusters

**FIGURE 3:**
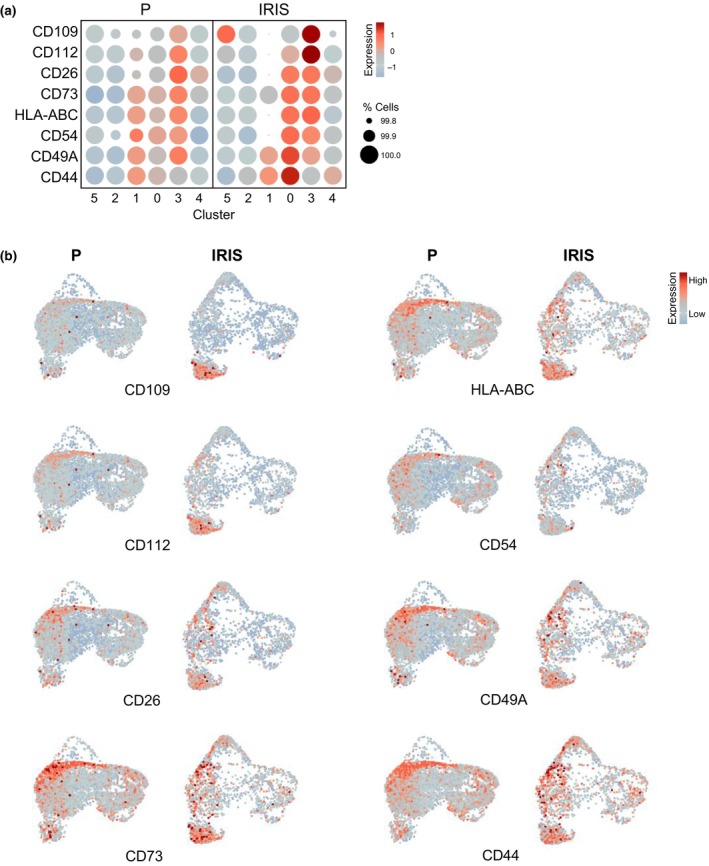
Surface proteins in cell clusters. (a) Levels of the studied cell‐surface proteins in each cluster of P and IRIS populations. Color indicates average expression scaled across all clusters and dot size represents the percentage of cells expressing specific ADTs. (b) Expression of surface proteins from (a) distributed in UMAP space for proliferating (P) and senescent (IRIS) cells.

We then examined the distribution and expression levels of cell‐surface proteins in each identified cluster, focusing specifically on their preferential presence in IRIS relative to P cells, both in aggregate (Figure 3a) and individually by UMAP analysis (Figure 3b). Remarkably, cluster 3 cells, representing senescent cells based on transcriptomic profiles (Figure 2e, Table S2), exhibited heightened expression levels of CD109 and CD112 proteins on the cell surface. The other surface proteins studied in this panel were also present in cluster 3 cells, albeit at lower levels, and their abundance did not significantly differ between IRIS and P cells. The selective presence of CD109 and CD112 in cluster 3 supports the notion that surface protein markers can be used to identify senescent cells, although other senescent cells (e.g., those in cluster 4) were not recognized by any of the surface proteins in this panel. Despite an overall decline in cluster 0 after IRIS, we observed notable increases in the levels of CD26, CD73, HLA‐ABC, CD54, CD49A, and CD44 in this cluster, indicating that specific cell‐surface proteins can be selectively present on the surface of certain subpopulations of senescent cells. These results support the notion that senescent cells exhibit a broad continuum of transcriptomic states, with some senescent cells displaying tractable cell‐surface markers.

### 2.4 Phenotypic features of senescent cell clusters

**FIGURE 4:**
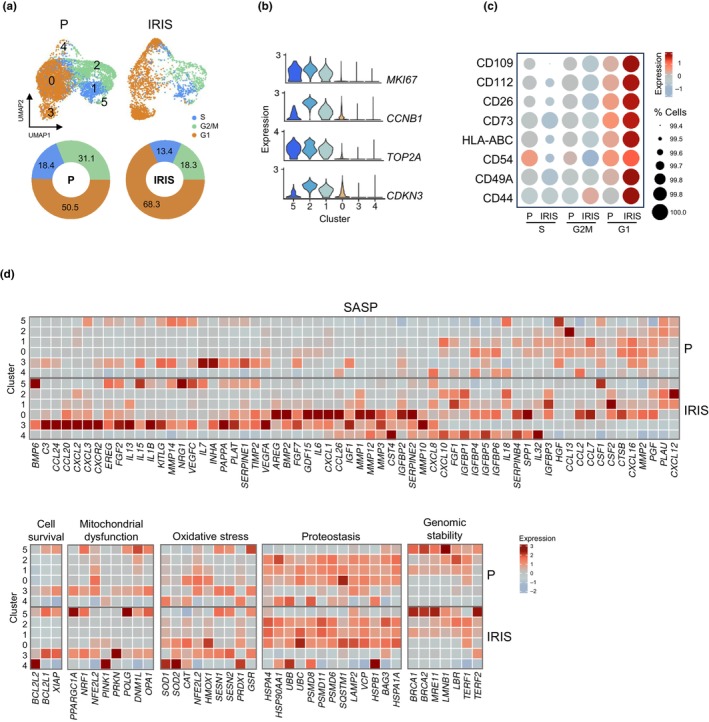
Functional assessment of cell clusters. (a) Cell cycle states (*top*) and percentage of cells in each phase (*bottom*) of P and IRIS cells assigned by the Seurat CellCycleScoring analysis. (b) Expression of mRNAs encoding specific cell cycle regulatory proteins in each cluster. (c) Cell‐surface protein levels in cell populations expressing transcriptomes consistent with G1, S, and G2/M phases. Color indicates scaled average expression and dot size represents the percentage of cells expressing specific ADTs. (d) Average expression of selected mRNAs encoding proteins representing the indicated hallmarks of senescence in each cluster of P and IRIS populations.

To confirm the senescent state of the cell clusters identified (Figure 2d), we compared their transcriptomes with those associated with different hallmarks of cellular senescence. First, considering that senescent cells display persistent growth arrest, we used the CellCycleScoring function in the Seurat package to determine where these cells distribute across the cell division cycle based on cell cycle‐phase‐specific transcriptomic signatures. As anticipated, cell cycle scoring estimated that ~70% of IRIS cells were in the G1 phase, while 50% of P cells were in the G1 phase (Figure 4a). Interestingly, cells in clusters 0, 3, and 4 were particularly enriched in G1 phase‐associated transcriptomes in IRIS populations. Accordingly, these clusters showed reduced levels of transcripts encoding key regulators of cell division, including *MKI67*, *CCNB1*, *TOP2A*, and *CDKN3* mRNAs (Figure 4b), further confirming that subsets of these cell populations were likely not dividing. Importantly, the surface proteins studied were markedly more abundant in G1 cells present in the IRIS group relative to G1 cells in the P population, indicating key differences in the G1 states between these two groups; S and G2/M phases had very low levels of the surface proteins (Figure 4c). These results strongly support the notion that the membrane markers identified are specifically present on the surface of senescent cells and can be used to selectively distinguish senescent cells.

Second, we focused on the secretory phenotype (SASP), another major hallmark of senescence, by using the SenMayo SASP dataset (Saul et al., 2022) (Figure 4d). Our analysis revealed positively enriched SenMayo gene set scores in clusters 3 and 0, with the enrichment being statistically significant in cluster 0. We observed great heterogeneity in transcriptomes encoding SASP factors across the clusters. Cluster 3 expressed high levels of transcripts encoding different interleukins and chemokines, while cluster 4 expressed high levels of transcripts encoding insulin‐like growth factor‐binding proteins. Cluster 0 displayed increased levels of mRNAs encoding chemokines CCL2 and CCL7, but also shared expression of multiple SASP factors with clusters 3 and 4, such as mRNAs encoding matrix metalloproteinases, serpins, and growth factors. In sum, the transcriptomes encoding SASP factors in the clusters suggest that various cell populations may contribute in different ways to the SASP, with some clusters potentially promoting inflammation and tissue damage, while others possibly playing a protective role in tissue repair and homeostasis.

We also evaluated the expression of several genes commonly associated with other hallmarks of cellular senescence (Saul et al., 2022) (Figure 4d). On the one hand, the cells of clusters 0, 3 and 4 consistently showed decreased levels of mRNAs encoding proteins involved in maintenance of genomic stability. On the other hand, these clusters expressed transcriptomes encoding a range of proteins involved in cell survival, mitochondrial dysfunction, oxidative stress, and cellular proteostasis, indicating a broad spectrum of senescent cell states and putative functions. Altogether, these findings indicate that the cells of clusters 0, 3, and 4 expressed transcriptomes highly enriched in G1‐phase proteins, yet each population exhibits different transcriptomic patterns of genes considered to be indicators of senescent phenotypes, suggesting the existence of dynamic differences and divisions of function following the initiation of senescence.

### 2.5 Subclustering reveals an additional subpopulation of senescent phenotype

The initial clustering revealed that cluster 0 included cells with transcriptomic similarities between P and IRIS populations, which was unexpected, as senescent cells typically form clusters distinct from those in proliferating cells. The transcriptomes within cluster 0 were distinctly associated with G1 phase cells, but also with a range of other traits (Figure 4) without distinct global trends (Figure 2e). At the same time, many surface proteins were recognized within cluster 0 cells after IRIS treatment (Figure 3), suggesting the existence of subclusters within this cluster. Thus, further subclustering of cluster 0 was carried out to investigate this heterogeneity.

**FIGURE 5:**
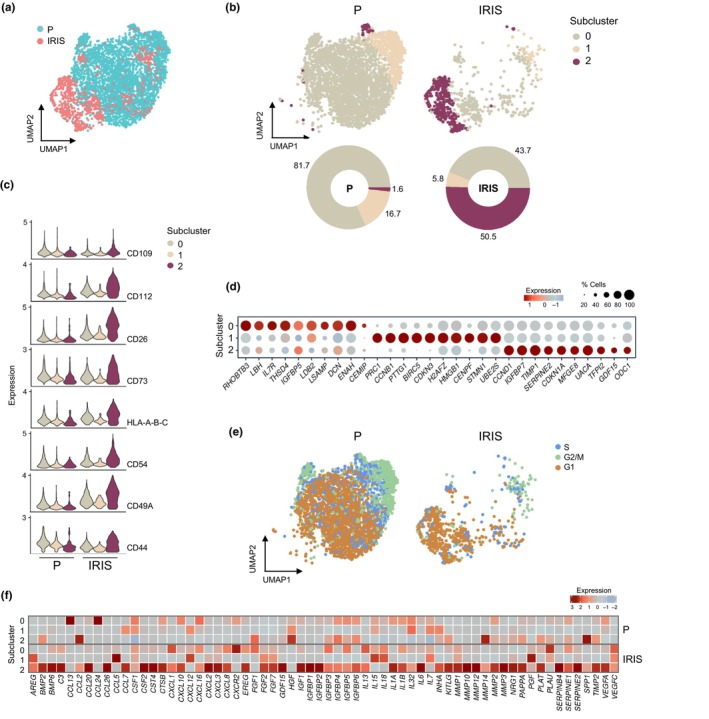
Subclustering of cluster 0 cells. (a) UMAP representation of the integrated cells of cluster 0 from Figure 2d, color‐labeled for P and IRIS cell populations. (b) UMAP illustrating the cell subclusters (*top*) and percent composition of each subcluster (*bottom*) in P and IRIS populations. (c) Expression levels of the cell‐surface proteins in each subcluster of P and IRIS populations. (d) Top ten highly expressed RNA markers of each subcluster. Color indicates average expression scaled across all subclusters, and dot size represents the percentage of cells expressing specific RNAs. (e) Cell cycle phase of P and IRIS cells assigned by the Seurat CellCycleScoring analysis. (f) Average expression of mRNAs encoding SASP factors in each subcluster of P and IRIS populations, using the SenMayo dataset as reference.

Subclustering analysis of this cluster in cells from P and IRIS groups (Figure 5a) distinguished three subclusters, representing three different cell states within cluster 0 (Figure 5b). Notably, the cells of subcluster 2 comprised more than 50% of all IRIS cells, showing increased presence of all the cell‐surface proteins studied (Figure 5c, Figure S3). The specific transcriptome of subcluster 2 included high expression of mRNAs encoding proteins frequently associated with senescence, such as CCND1, IGFBP7, TIMP1, SERPINE2, CDKN1A, and GDF15 (Figure 5d, Figure S4a, Table S3). In addition, subcluster 2 cells were predominantly in G1, as assessed by cell cycle phase scoring (Figure 5e), and had increased mRNAs encoding SASP factors and proteins involved in cell survival and the response to oxidative stress (Figure 5f; Figure S4b), confirming that subcluster 2 cells from cluster 0 were senescent. It is important to note that subcluster 0 of cluster 0, comprising 81.7% of the P population, also comprised primarily G1‐phase cells (Figure 5b,e); however, subcluster 0 cells did not display high levels of the surface proteins nor SASP factor‐encoding mRNAs, supporting the view that these cells are simply progressing through G1 but are not senescent. Importantly, these findings underscore the value of using surface proteins to identify senescent cells.

In closing, we noted a discrepancy between the levels of surface proteins and the levels of the mRNAs encoding these proteins, which were often rather low and difficult to detect (Figures S5 and S6). The surface proteins alone were easier to detect and displayed more consistent expression patterns (Figure S3). In summary, the cell‐surface proteins used in CITE‐seq analysis enabled us to more precisely define subpopulations of senescent cells that were not sufficiently distinguished based on the primary transcriptomic clustering. This work underscores the value of studying the cell surfaceome, in combination with the transcriptome, to identify senescent cell populations.

## 3 DISCUSSION

The accumulation of senescent cells during aging has been linked to the decline of organ and tissue function that characterizes aging physiology and leads to age‐related disease (Faulk et al., 2023; McHugh & Gil, 2018). The vast heterogeneity of senescent cells poses substantial challenges towards understanding the mechanisms that govern senescence in order to develop effective interventions (Kirschner et al., 2020; Kwiatkowska et al., 2023). Changes in the type and abundance of proteins on the surface of the senescent cell may help the cell modify its interaction with the microenvironment. For example, CD95 (also known as FAS) delays the establishment of senescence in mouse embryonic fibroblasts, as evidenced by the fact that CD95 ablation elevated markers of senescence including SA‐β‐Gal activity, and the proteins p16, p21, and IL6 (Saha et al., 2023).

We identified senescence‐associated surface proteins by mass spectrometry analysis in lung fibroblasts rendered senescent by both RS and IRIS. For IRIS, we exposed cells to 10 Gy IR and incubated them for an additional 10 days, a widely used strategy to induce senescence (Meng et al., 2021; Mikula‐Pietrasik et al., 2020). This analysis revealed both increased and decreased proteins, suggesting the existence of dynamic changes in the landscape of cell‐surface proteins in senescence. Among the elevated surface proteins that were shared between the two models of senescence (Figure 1), we focused on CD49A, CD73, CD109, CD54, CD112, HLA‐ABC, CD44, and CD26; these proteins were chosen because (i) CITE‐seq antibodies specific for these proteins were available for this study, (ii) they were preferentially enriched in senescence, and (iii) they have important biological functions linked to senescence. CD49A, a member of the integrin family, is involved in cell adhesion and migration, promoting tissue integrity and remodeling (Reilly et al., 2020; Siegers, 2018). CD73 is responsible for the generation of extracellular adenosine, which acts as a signaling molecule involved in physiologic processes including immune regulation and tissue protection (Minor et al., 2019; Rosemblatt et al., 2021). CD109 is a protein anchored by glycosylphosphatidylinositol that modulates transforming growth factor‐beta (TGF‐β, TGFB) signaling, thereby regulating cell proliferation, differentiation, and immune responses (Bizet et al., 2011; Tanabe et al., 2022). CD54, also known as intercellular adhesion molecule‐1 (ICAM1), plays a crucial role in leukocyte adhesion and migration during inflammatory responses (Bui et al., 2020). CD112, also called nectin‐2, is a cell adhesion molecule involved in the formation of adherens junctions and in the regulation of cell–cell interactions (Duraivelan & Samanta, 2020). HLA‐A, ‐B, and ‐C, antigens of major histocompatibility complex class I, are responsible for presenting antigens to cytotoxic T cells, playing a crucial role in immune recognition and response (Kedzierska & Koutsakos, 2020). CD44 is a glycoprotein involved in cell–cell and cell‐matrix interactions, and participates in processes such as cell adhesion, migration, and signaling (Chen et al., 2018). CD26, also known as dipeptidyl peptidase‐4 (DPP4), is an enzyme involved in the regulation of physiological processes such as immune modulation, metabolism, and inflammation (Klemann et al., 2016).

We characterized senescent cells by CITE‐seq analysis using a cocktail of barcoded antibodies to label and quantify the aforementioned cell‐surface proteins and to study them alongside the endogenous transcriptomes at the single‐cell level. Our primary analysis revealed six clusters (Figure 2d); one of these clusters (cluster 3) was transcriptomically characterized as a senescent‐cell population, presenting high levels of surface proteins CD109 and CD112 after IR‐induced senescence (Figure 3). Cell cycle scoring uncovered three clusters (0, 3, and 4) enriched in G1‐phase cells whose transcriptomes encoded the vast majority of SASP factors and other hallmark factors of senescence, but the transcript profiles were different for each cluster (Figure 4a). The increased presence of the surface proteins, particularly in the IRIS group (Figure 4c), allowed us to distinguish G1 cells that were senescent due to IRIS from those that were proliferating and just transiting through the G1 phase. Subsequent subclustering of cells in cluster 0 revealed additional senescent cell subpopulations; subcluster 2, in particular, showed increased levels of cell‐surface proteins present in IR‐treated cells (Figure 5). Subcluster 2 also expressed increased levels of mRNAs encoding well‐established indicators of senescence such as CDKN1A, GDF15 or TIMP1 and SASP factors. Collectively, these findings highlight the striking heterogeneity of senescent cells, even though senescence was triggered in a single cell type (fibroblasts) using a single damaging agent (IR).

CITE‐seq has been used to study different diseases, including immune‐related disorders, influenza, and COVID‐19 (Lakkis et al., 2022), often using fixed, permeabilized tissues, which enabled the detection of intracellular proteins. In combination with spatial transcriptomic analysis, CITE‐seq analysis revealed distinct immune reactions in different tissues to identify intracellular proteins implicated in the response to COVID‐19 mRNA vaccines (Liu et al., 2023). Machine learning algorithms were applied to analyze CITE‐seq datasets, leading to the discovery of proteins and mRNAs important for classifying cell types in the spleen and lymph nodes (Li et al., 2022). In other studies, CITE‐seq analysis was applied to bridge the mRNA‐protein gap in the characterization of human breast cancers and melanoma (Wu et al., 2021) and enabled the identification of immune cell subsets, which is often challenging to discern only through transcriptomic signatures.

Our study was limited by the availability of CITE‐seq antibodies (Figure 1, Table S1). Antibodies required for more comprehensive profiling of additional surface proteins associated with senescence, including antibodies recognizing posttranslationally modified surface proteins, are unavailable at present. Despite the high cost of the CITE‐seq methodology relative to scRNA‐seq analysis alone, future studies should include a wider range of antibodies directed at senescence surface proteins and posttranslationally modified surface proteins to identify additional clusters and further subclassify senescent cells. Future studies should also focus on further identifying surface proteins associated with other cell types and senescence paradigms outside of fibroblasts and IRIS. It will be of particular interest to carry out CITE‐seq analysis over time to identify dynamic changes in the cell‐surface protein landscapes of senescent cells, both in culture and in tissues. For example, future studies should study shorter culture times to appreciate the early dynamics of senescence‐associated surface proteins, as well as extend the observation periods in cultured cells to examine the long‐term effects of senescence (Schafer et al., 2017). We previously studied mouse senescent cells in atherosclerotic plaques using the surface marker CD26 (Herman et al., 2023); following this approach, in vivo CITE‐seq models will allow us to observe the effects of senescence in tissues, potentially uncovering tissue‐specific and systemic responses to different senescence inducers, including damaging stressors and aging.

Another key limitation of CITE‐seq is that access to intracellular epitopes, such as p16 or p53, in intact cells, is not currently possible. Thus, advances in the CITE‐seq technology to internalize barcoded antibodies without disrupting the cell membrane will be extremely valuable. Adaptations of CITE‐seq to analyze intracellular tags (Stoeckius et al., 2017) as well as chromatin at the single‐cell level, in conjunction with transcriptomic analysis in the same cell, will be particularly informative.

To leverage its broader applicability, the CITE‐seq methodology should include both a substantially larger array of CITE‐seq antibodies and a wider range of senescence models, such as those induced by chemotherapy, dietary factors, and aging. It will be especially interesting to employ CITE‐seq to discern the heterogeneity and dynamics of senescent cells in tissues.

As we report here, targeting surface proteins can help identify and classify senescent cell subpopulations, providing a foundational catalog that can be expanded by incorporating various senescence triggers, cell types, and species. Our laboratory has begun to develop custom CITE‐seq antibodies for cell‐surface proteins in mouse. Although producing these antibodies is costly and time‐consuming, they are essential for advancing our understanding and therapeutic targeting of detrimental subpopulations of senescent cells in animal models.

In summary, we have shown proof‐of‐concept evidence that CITE‐seq analysis can identify different senescence programs and offer a more comprehensive view of senescence heterogeneity. Detailed knowledge of specific senescent subpopulations will be critical for guiding the development of targeted therapeutic strategies that mitigate disease processes exacerbated by aberrant senescence.

## 4 METHODS

### 4.1 Cell culture, senescence induction, and SA‐β‐gal activity

Human diploid WI‐38 fibroblasts (Coriell Institute for Medical Research: ID AG06814) were cultured in Dulbecco's modified Eagle's medium (DMEM, Gibco) supplemented with 20% FBS, 1% antibiotics, and 1% nonessential amino acids (Gibco). Cell cultures were maintained in an incubator at 37°C and 5% CO_2_. To reach replicative senescence (RS), cells were cultured until replicative exhaustion [reaching population doubling level (PDL) ~54]. To trigger ionizing radiation (IR)‐induced senescence (IRIS), proliferating (PDL24) fibroblasts were exposed to 10 Gray (Gy) and cultured for an additional 10 days. Senescence was confirmed by assaying senescence‐associated β‐galactosidase (SA‐β‐Gal) activity following the manufacturer's instructions (Cell Signaling Technology). Briefly, cells were washed with 1× PBS, fixed for 10 min at room temperature, and stained overnight with a freshly prepared X‐Gal solution (pH 6.0). Micrographs were acquired using Nikon Digital Sight camera adapted to a microscope (Nikon Eclipse TS100).

### 4.2 Surface protein biotinylation and isolation

Surface proteins were biotinylated using the Pierce™ Cell Surface Biotinylation (EZ‐Link Sulfo‐NHS‐SS‐Biotin) and Isolation Kit (Thermo Fisher Scientific, A44390) following the manufacturer's protocol. Briefly, cell pellets of three to five million cells were resuspended and labeled with 0.25 mg/mL Sulfo‐NHS‐SS‐Biotin for 10 min at 25°C. Cells were centrifuged at 5000 × g for 3 min at 4°C and washed using ice‐cold Tris Buffered Saline (TBS). Biotin‐labeled cells were lysed on ice for 30 min, and NeutrAvidin Agarose was used to collect the tagged proteins from the lysates. Dithiothreitol (DTT) was employed in the elution solution to break down the disulfide bonds of the biotin label. Cell surface proteins were isolated for mass spectrometry (MS) analysis (performed in duplicate) and western blot analysis (performed in triplicate).

### 4.3 Mass spectrometry‐based proteomics of surface proteins

Cell surface proteins were identified in WI‐38 fibroblasts that were proliferating (~PDL24) or rendered senescent by IRIS or RS (~PDL54). Mass spectrometry analysis (by Poochon Scientific) was performed by liquid chromatography‐coupled tandem MS (LS‐MS/MS) using a Q‐Exactive hybrid quadrupole orbitrap mass spectrometer and Nano‐EasySpray Ion Source (Thermo Fisher Scientific). Raw data files acquired from each sample were searched against the human protein sequences database and UniprotKB/Swiss‐Prot database using the Proteome Discoverer (v1.4) based on the SEQUEST algorithm (Thermo Fisher Scientific). The minimum peptide length specified was 5 amino acids, and the maximum false peptide discovery rate (FDR) was specified as 0.01. All assembled proteins with peptide spectrum match (PSM) counts were quantified and normalized using the normalized spectral abundance factors (NSAFs) to assign their relative abundance. MS data were deposited to MassIVE MSV000093336, doi:10.25345/C5639KG5J.

### 4.4 Western blot analysis

Whole‐cell lysates (input) and surface proteins were prepared using the Pierce™ Cell Surface Biotinylation (EZ‐Link Sulfo‐NHS‐SS‐Biotin) and Isolation Kit (Thermo Fisher Scientific, A44390) following the manufacturers' instructions. After surface biotinylation, cells were lysed, and total protein concentration was measured by the Pierce BCA Protein Assay Kit (Thermo Fisher Scientific). A fraction of the whole‐cell lysate (10%) was saved as input, the remaining 90% was used to isolate labeled surface proteins. After adding Laemmli sample buffer (Bio‐Rad, #1610747), we performed western blot analysis by separating input and surface samples on 4–12% SDS‐containing polyacrylamide gels under reducing conditions and transferred onto nitrocellulose membranes (Bio‐Rad). Membranes were blocked in 5% nonfat dry milk for 1 h at room temperature and incubated at 4°C overnight with primary antibodies diluted in blocking solution. Primary antibodies from Cell Signaling Technology recognized CD49A/ITA1 (#15574), CD73/NT5D (#13160), CD109 (#24765), CD54/ICAM1 (#67836), CD112/NECT2/PVRL2 (#95333), CD44 (#37259), and CD26/DPP4 (#67138); primary antibodies from Santa Cruz Biotechnology recognized HLA A/B/C (sc‐52810) and ACTB (β‐Actin; sc‐47778). After incubation with the appropriate secondary antibodies for 1 h at room temperature, the protein signals were visualized using an enhanced chemiluminescence (ECL) solution (Kindle Biosciences, R1002); the digitized images were captured using a ChemiDoc MP Imaging system (Bio‐Rad Laboratories). Western blot analyses were performed in triplicate.

### 4.5 Labeling cells with barcoded antibodies, single‐cell library construction, and RNA‐sequencing (RNA‐seq) analysis

WI‐38 cells (PDL24) were either left untreated (Proliferating, “P”) or were treated with IR (10 Gy) to induce IRIS. Ten days later, cells were detached using TrypLE Express™ (Gibco #12605–010) and 1–2 million cells were resuspended in 50 μL Cell Staining Buffer (BioLegend #420201) with 10% FBS. Cells were blocked by the addition of 5 μL Human TruStain FcX™ Fc Blocking reagent (BioLegend, #422301) for 10 min at 4°C and an antibody pool was prepared containing 1 μg of each TotalSeq™ B antibodies from BioLegend (CD112 #337425, HLA‐A,B,C #311451, CD44 #103071, CD54 #353135, CD26#302727, CD49A #328323, CD73 #344033, and CD109 #323311). After bringing the final volume to 50 μL with Cell Staining Buffer and centrifugation at 14,000 × g for 10 min at 4°C, the antibody mixture was added to the cells and incubated at 4°C for 30 min. Cells were then washed with 1 mL of Cell Staining Buffer, centrifuged at 400 × g for 5 min at 4°C, and then resuspended in 1 mL of PBS supplemented with 5 mM EDTA for a final concentration of 10^6^ cells/mL. Antibody‐labeled cells were then stained with 2 μg/mL of propidium iodide for 10 min at 25°C in the dark, and 16,000 viable cells were sorted using WOLF G2 (Nanocellect Biomedical, Inc.). Sorted cells were spun down at 500 × g for 15 min and the resulting cell pellet was resuspended in PBS; the cell suspension was then used for single‐cell RNA‐sequencing (scRNA‐seq) analysis with Feature barcoding technology for cell surface proteins using 10× Genomics technology.

Single‐cell libraries were prepared using Chromium Next GEM Single Cell 3′ Kits v3.1 with the 3′ Feature Barcode kit using Chip G (10× Genomics) following the manufacturer's protocol. Briefly, cells were loaded onto the Chromium Controller Instrument for generation of Gel Beads‐in‐emulsion (GEMs), the captured cells were lysed, and the RNA was barcoded while reverse‐transcribed in each GEM. GEMs were subsequently broken, and the synthetized cDNA used for library preparation. cDNA quality was assessed on the Agilent Bioanalyzer with the high‐sensitivity DNA kit (Agilent Technologies), whereas the final library products were assessed on Agilent TapeStation using the Agilent D1000 ScreenTape assay. The libraries were sequenced on an Illumina NovaSeq 6000 sequencer with 70,000–120,000 reads per cell. RNA‐seq data were deposited in GEO (GSE250041).

### 4.6 CITE‐seq data analysis

CITE‐seq data encompassing both RNA and ADT (Antibody‐Derived Tags) libraries were initially processed using the Feature barcoding workflow pipeline within Cell Ranger version 6.1.1 by 10× Genomics. The data were subsequently aligned to the human GRCh38 reference genome. The resulting read count matrices were further subjected to analysis in R, employing the Seurat package, version 4.1.0 (Hao et al., 2021). To ensure the downstream analysis was of high quality and excluded potential doublets, a rigorous cell quality filtering process was implemented for each sample. This filtering step removed cells that expressed fewer than 300 or more than 12,000 transcripts, as well as cells with fewer than 2000 or more than 150,000 RNA counts. Additionally, cells containing more than 10% mitochondrial RNAs and those with over 20,000 ADT counts were excluded. After filtering, a total of 8093 cells were used for downstream analysis. Furthermore, transcripts detected in fewer than three cells were omitted from the analysis. Normalization of RNA counts was conducted using the “LogNormalize” method, while the ADT data were normalized using “CLR” method. The top 2000 most variable genes were selected with the function “FindVariableFeatures.” Subsequently, integration anchors were obtained by running “FindIntegrationAnchors” and the samples were integrated with the function “IntegrateData.” After performing principal component analysis (PCA), the top 20 Principal Components determined by “ElbowPlot” method were used to create the Uniform Manifold Approximation and Projection (UMAP) with a resolution of 0.2 and distinguish cell clusters. Cell cycle phases were assessed using the “CellCycleScoring” function. The “FindAllMarkers” function in Seurat was employed to identify differentially expressed marker genes for each cluster.

## AUTHOR CONTRIBUTIONS

KA conceived the study and designed experiments; KA, KMM, QM, RM, DT, MR, JM, JF, YP, SD performed and analyzed experiments; HY and IB contributed intellectually and provided technical support; KA, KMM, MG wrote the manuscript.

## FUNDING INFORMATION

This work was funded by grant Z01‐AG000511, from the National Institute on Aging Intramural Research Program, National Institutes of Health.

## CONFLICT OF INTEREST STATEMENT

None declared.

## Supporting information


Appendix S1.

Table S1.

Table S2.

Table S3.

## Data Availability

The data that support the findings of this study are available from the corresponding author upon reasonable request.
