# Supplementary material for: Identification of senescent cell subpopulations by CITE‐seq analysis
Source: Aging Cell. 2024 Aug 14;23(11):e14297. doi: 10.1111/acel.14297 (PMC11561699; doi:10.1111/acel.14297)
Supplement: Supplementary file 1 — Appendix S1. [file ACEL-23-e14297-s002.pdf]

LEGENDS OF SUPPLEMENTARY FIGURES

Supplementary Figure S1. WI-38 fibroblasts were rendered senescent by culture through replicative exhaustion (PDL54) or by exposure to 10 Gy ionizing radiation (IR) and culture for 10 days (as in Figure 1a). After RNA isolation, RT-qPCR analysis was used to measure markers that increase (*p21*, *CXCL1*, *GDF15*, *BAFF*, *DPP4*, and *IL1A* mRNAs, as well as the long noncoding RNA *PURPL*) or decrease (*MKI67* and *LMNB1* mRNAs) with senescence.

Supplementary Figure S2. Validation of senescent clusters. (a) Expression patterns and levels of senescence-associated long noncoding RNAs (*NEAT* and *MEG3*) upregulated in cluster 3 and *CCND1* and *CDKN2A* mRNAs upregulated in cluster 4, in proliferating (P) and senescent (IRIS) cells. (**b, c**) Correlation of RNA expression (adjusted p-value <0.05) between (**b**) cluster 3 from the current study and cluster 5 from Wechter et al., 2023; and (**c**) cluster 4 from the current study and cluster 3 from Wechter et al., 2023.

Supplementary Figure S3. Expression patterns and levels of studied cell surface proteins in cluster 0 cells of P and IRIS populations. Cells bearing surface markers as determined by the presence of ADT are indicated.

Supplementary Figure S4. Validation of senescent subcluster 2 in cluster 0. (a) Expression patterns and levels of senescence-associated mRNAs upregulated in subcluster 2, in proliferating (P) and senescent (IRIS) cells. **(b)** Average expression of selected mRNAs encoding proteins representing indicated hallmarks of senescence in each subcluster of cluster 0, in P and IRIS populations.

Supplementary Figure S5. Expression patterns and levels of mRNAs corresponding to cell-surface proteins in cluster 0 cells of P and IRIS populations. Cluster 0 cells expressing mRNAs corresponding to the surface markers identified by ADT are indicated.

Supplementary Figure S6. Expression patterns and levels of mRNAs corresponding to studied cell surface proteins in P and IRIS populations. In all clusters, cells expressing mRNAs corresponding to the surface markers identified by ADT are indicated.

LEGENDS OF SUPPLEMENTARY TABLES

Supplementary Table S1. Proteomic analysis of senescence-associated surface proteins (P vs. RS, and P vs. IRIS).

Supplementary Table S2. Marker genes representing differentially expressed transcriptomes in each cluster versus all other clusters.

Supplementary Table S3. Marker genes representing differentially expressed transcriptomes in each subcluster versus all other subclusters of cluster 0.

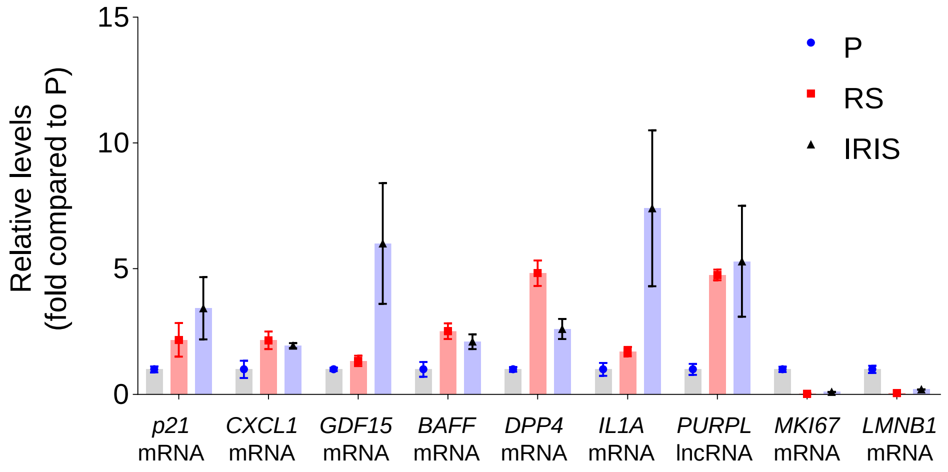
15
10
5
0
Relative levels
(fold compared to P)
P
RS
IRIS
p21 mRNA
CXCL1 mRNA
GDF15 mRNA
BAFF mRNA
DPP4 mRNA
IL1A mRNA
PURPL lncRNA
MKI67 mRNA
LMNB1 mRNA

(a)

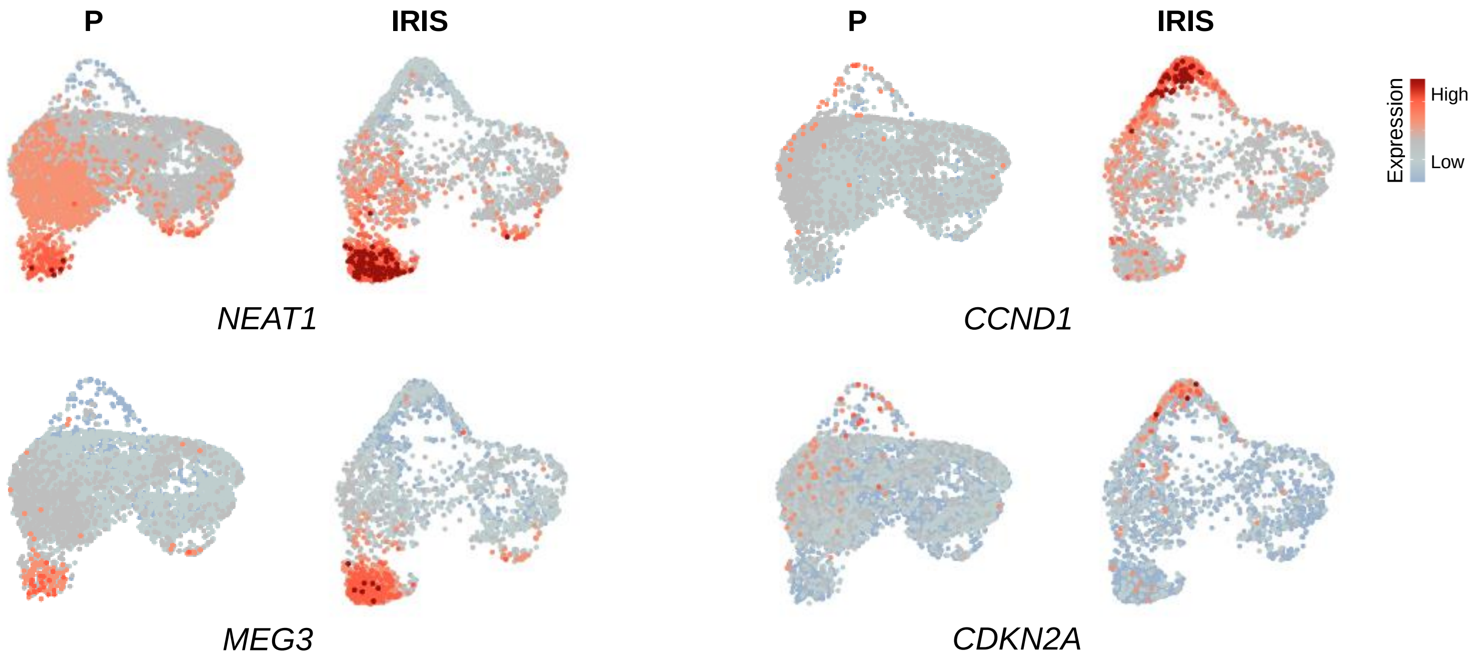
P
IRIS
P
IRIS
Expression
High
Low
NEAT1
CCND1
MEG3
CDKN2A

(b)

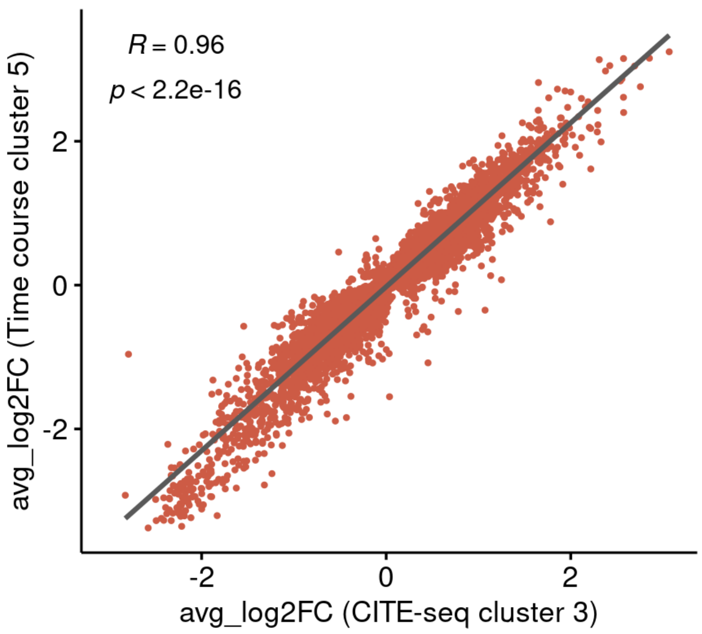
R = 0.96
p < 2.2e-16
avg_log2FC (Time course cluster 5)
avg_log2FC (CITE-seq cluster 3)
2
0
-2

(c)

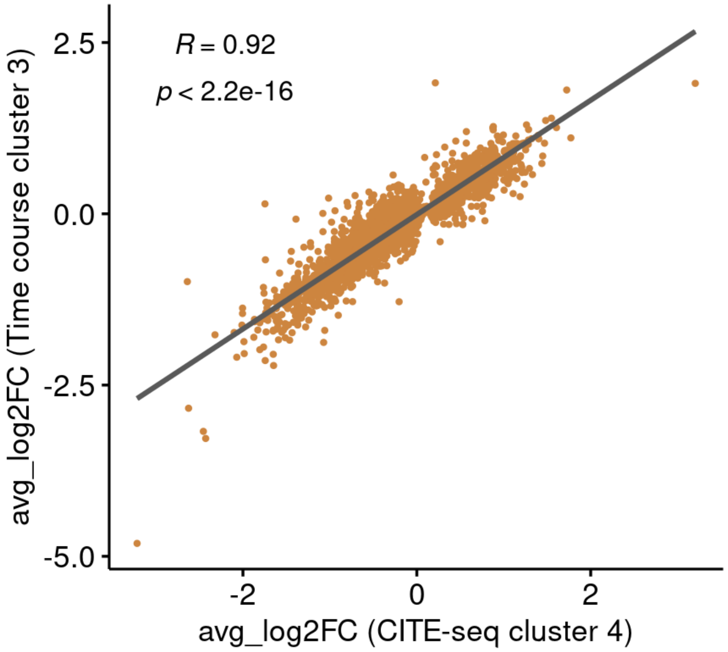
R = 0.92
p < 2.2e-16
avg_log2FC (Time course cluster 3)
avg_log2FC (CITE-seq cluster 4)
2.5
0.0
-2.5
-5.0
-2
0
2

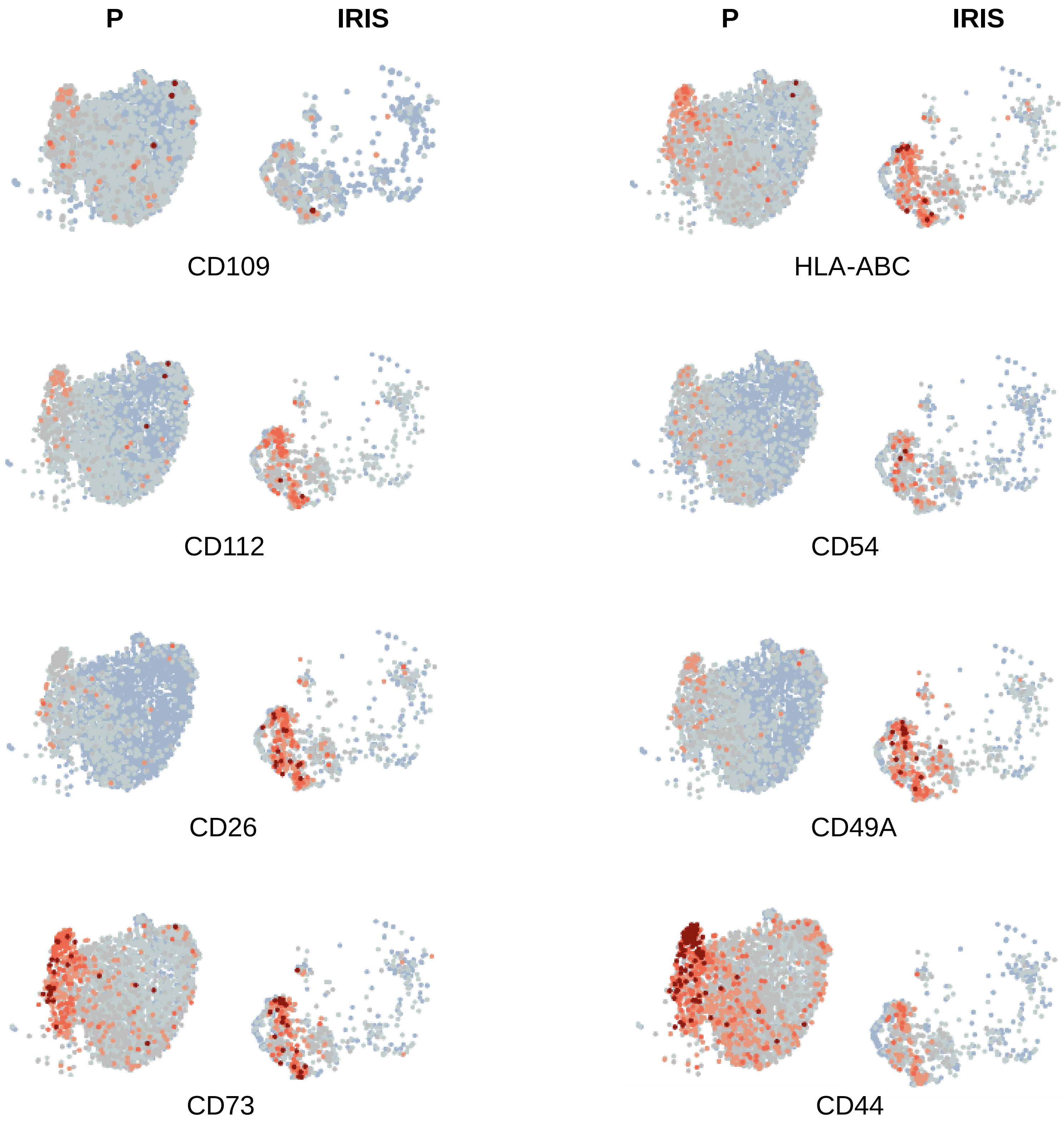
P
IRIS
P
IRIS
CD109
HLA-ABC
CD112
CD54
CD26
CD49A
CD73
CD44

(a)

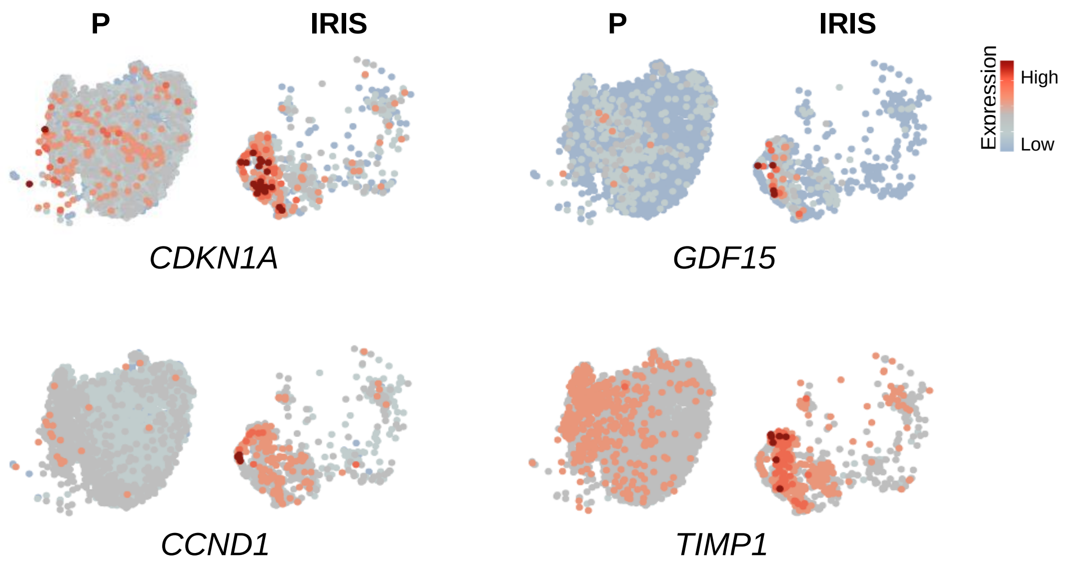
P
IRIS
P
IRIS
Expression
High
Low
CDKN1A
GDF15
CCND1
TIMP1

(b)

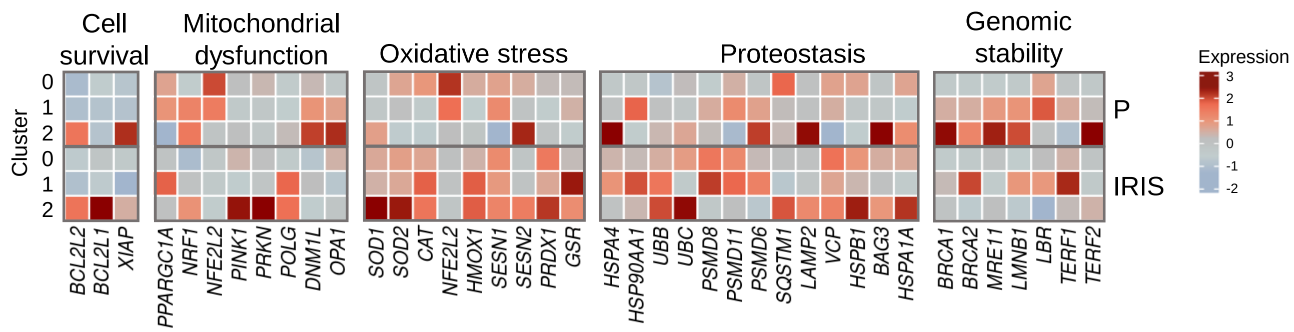
Cell survival
Mitochondrial dysfunction
Oxidative stress
Proteostasis
Genomic stability
Cluster
0
1
2
0
1
2
P
IRIS
Expression
3
2
1
0
-1
-2
BCL2L2
BCL2L1
XIAP
PPARGC1A
NRF1
NFE2L2
PINK1
PRKN
POLG
DNM1L
OPA1
SOD1
SOD2
CAT
NFE2L2
HMOX1
SESN1
SESN2
PRDX1
GSR
HSPA4
HSP90AA1
UBB
UBC
PSMD8
PSMD11
PSMD6
SQSTM1
LAMP2
VCP
HSPB1
BAG3
HSPA1A
BRCA1
BRCA2
MRE11
LMNB1
LBR
TERF1
TERF2

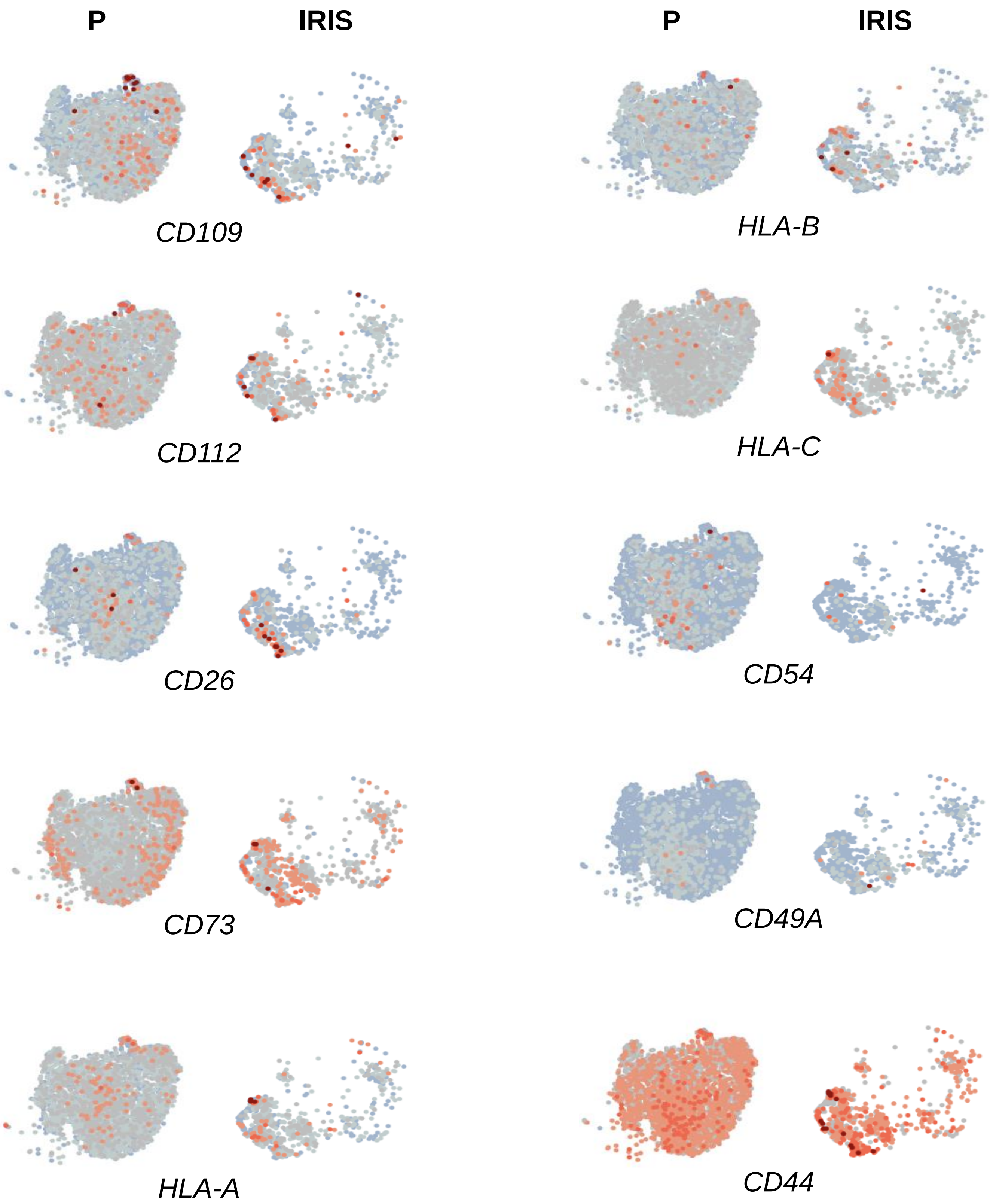
P
IRIS
P
IRIS
CD109
HLA-B
CD112
HLA-C
CD26
CD54
CD73
CD49A
HLA-A
CD44

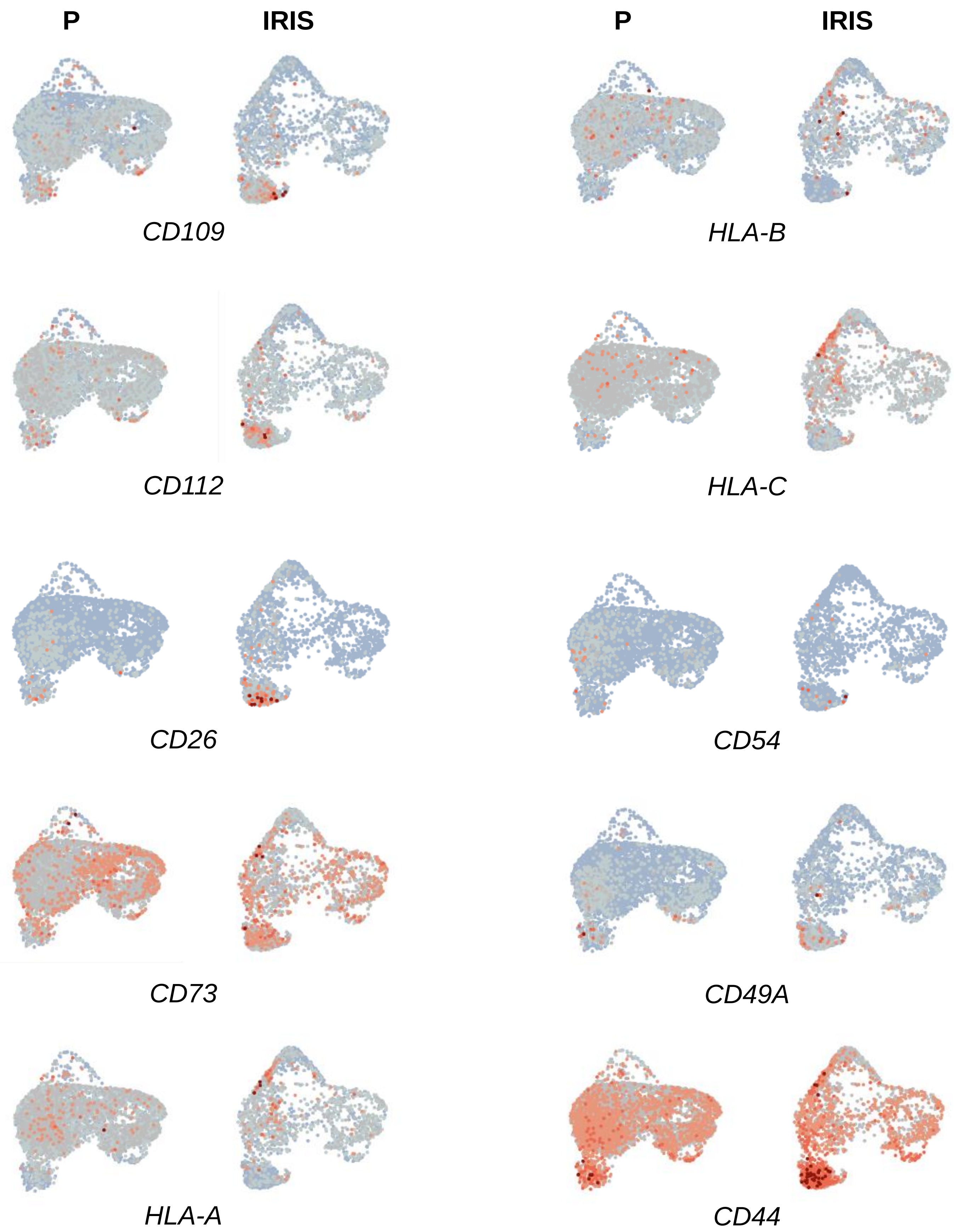
P
IRIS
P
IRIS
CD109
HLA-B
CD112
HLA-C
CD26
CD54
CD73
CD49A
HLA-A
CD44
